# Type VIII collagen: advances in matrix biology and translational promise

**DOI:** 10.3389/fbioe.2025.1732988

**Published:** 2026-01-20

**Authors:** Haiyan Shi, Yufeng Yu, Kaixuan Guo, Rongli He

**Affiliations:** 1 Shanxi Key Laboratory of Functional Proteins, School of Basic Medical Sciences, Shanxi Medical University, Taiyuan, China; 2 Shanxi Jinbo BioPharmaceutical Co., Ltd., Taiyuan, China

**Keywords:** angiogenesis, clinical translation, extracellular matrix, pathophysiology, type VIII collagen

## Abstract

Type VIII collagen, a member of the short-chain collagen family, plays essential roles in structural support, functional regulation, and mechanobiology across multiple organ systems. Although early studies suggested ubiquitous expression, emerging single-cell transcriptomic and proteomic analyses have refined this view, demonstrating selective enrichment in corneal endothelial cells, vascular smooth muscle cells, activated fibroblasts, and tumor-associated extracellular matrix (ECM) compartments. These findings establish type VIII collagen as both a structural constituent of the ECM and a dynamic regulator of cell behavior. Functionally, type VIII collagen is critical for endothelial cell stability, angiogenesis, ECM remodeling, and mechanosignaling. Dysregulation of *Col8a1* and *Col8a2* is implicated in a broad spectrum of disorders, including vascular remodeling, tissue fibrosis, diabetic nephropathy, cancer progression, and corneal endothelial dystrophies. With growing mechanistic insight, translational applications are rapidly expanding. Current directions include gene-editing strategies targeting *Col8a2* for Fuchs’ endothelial corneal dystrophy, RNA-based approaches to dissect *Col8a1*and *Col8a2* regulation in fibrotic and vascular disease, and the development of biomaterials incorporating type VIII collagen–derived motifs to promote endothelial repair and guide angiogenesis. Moreover, its restricted expression profile supports its potential utility as a diagnostic and prognostic biomarker. Collectively, these advances position type VIII collagen as a multifunctional ECM regulator with substantial promise for disease diagnostics, therapeutic innovation, and biomaterial engineering.

## 1 Introduction

Collagen is a natural macromolecular protein and one of the most important biopolymers in biomaterials research, accounting for approximately 25%–30% of the total protein content of the human body, with reported variation between sexes. Recent extracellular matrix–focused proteomic analyses further indicate that collagen density in male skin and connective tissue is generally higher than in females (Keaney, 2016). Structurally, unlike most proteins that form α-helices, collagen adopts a unique and highly stable triple-helical configuration composed of three intertwined polypeptide chains. (Shoulders and Raines, 2009). As the most abundant structural protein in mammals (Ricard-Blum, 2011), collagen is essential for maintaining the biomechanical integrity and functional organization of the extracellular matrix (ECM) (Zhou H. et al., 2024). According to updated genomic annotations, the human genome encodes 28 distinct collagen types arising from 45 functional collagen genes (Martin et al., 2023). Among these, type VIII collagen is a member of the short-chain, non-fibrillar collagen family. Although it is widely distributed in specialized ECM niches, including the post-elastic layer, vascular basement membranes, and renal glomeruli, its biological relevance has long been underestimated relative to classical fibrillar collagens. This knowledge gap has largely resulted from its low tissue abundance, technical challenges in isolating intact trimers, and the previous lack of high-resolution structural data for the full-length molecule.

Recent advances, however, have substantially renewed interest in type VIII collagen. Structural elucidation of its NC1 domain has revealed previously unrecognized determinants of trimer stability and has identified candidate interfaces responsible for higher-order lattice assembly. In addition, accumulating evidence indicates that (Li et al., 2024), type VIII collagen modulates integrin αvβ5 signaling, particularly in corneal endothelial cells, providing the first molecular-level insight into its role in regulating cell polarity, cytoskeletal organization, and barrier function. Together with genetic studies linking *Col8a1* and *Col8a2* mutations to vascular remodeling, Fuchs’ endothelial corneal dystrophy and diabetic corneal endothelial dysfunction, these findings have significantly advanced understanding of the functional importance of type VIII collagen. Continued investigation of this molecule will not only clarify its roles in physiology and disease, but also support the development of novel strategies in tissue engineering, regenerative medicine, and targeted therapy.

## 2 Structure and distribution of type VIII collagen

### 2.1 Structure of type VIII collagen

Type VIII collagen is a member of the short-chain, non-fibrillar collagen family. It was first isolated in the 1980s by Helene Sage and colleagues from the culture medium of adult bovine aortic endothelial cells and was initially termed endothelial collagen (Sage et al., 1980). The protein is composed of two genetically distinct α chains, α1 (*Col8a1*) and α2 (*Col8a2*), each with an approximate molecular mass of 60 kDa (Loeffler et al., 2011). Subsequent studies demonstrated that type VIII collagen can assemble into discontinuous triple-helical molecules in cultured proliferating cells, with a major chain molecular weight of approximately 180 kDa (Kapoor et al., 1988). Type VIII collagen exhibits notable trimeric heterogeneity (Mann et al., 1990). In addition to the conventional α1/α2 heterotrimers, it can also form homotrimers composed exclusively of either α1 or α2 chains (Rosenblum, 1996; Greenhill et al., 2000), a feature that likely contributes to its functional diversity. Both *Col8a1* and *Col8a2* are expressed across multiple cell types (Sage et al., 1980; Paulus et al., 1991; Benya, 1980; Sage et al., 1983; Sage et al., 1984; Benya and Padilla, 1986; Rüger et al., 1994; Adiguzel et al., 2013). The full-length human *Col8a1* and *Col8a2* proteins consist of 744 and 704 amino acids, respectively (Ma et al., 2012). The *Col8a1* gene is located on chromosome 3, whereas *Col8a2* resides on chromosome 1. Structurally, each α chain contains a short collagenous triple-helix domain flanked by a C-terminal non-collagenous domain (NC1) and an N-terminal non-collagenous domain (NC2) (Muragaki et al., 1991). Structural information on type VIII collagen remains incomplete. Currently, only crystal structures of the NC1 domain are available in the Protein Data Bank (PDB: 1O91), whereas the full-length triple-helical structure has not been resolved, likely due to technical challenges associated with crystallizing flexible collagenous regions. Analysis of the trimeric crystal structure of the murine NC1 domain reveals that it belongs to the C1q-like protein family and forms a stable homotrimer. The surface of this domain contains bands enriched in aromatic residues that are thought to facilitate supramolecular assembly. Notably, unlike type X collagen, the NC1 domain of type VIII collagen lacks characteristic calcium-binding clusters, suggesting a distinct assembly mechanism (Kvansakul et al., 2003). Functionally, the NC1 domain is critical for trimer stabilization and contributes to basement membrane organization.

### 2.2 *In vivo* distribution of type VIII collagen

Type VIII collagen is widely distributed across human tissues. Early immunohistochemical investigations (Kittelberger et al., 1990) described it as “ubiquitously expressed with limited tissue specificity,” based on its detection in diverse basement membranes and connective tissues, including the descemet membrane of the corneal endothelium, sclera, choroid, optic nerve sheath, tendon, skin, periosteum, perichondrium, and glomerular mesangium (Altenbach et al., 1990; Franzke et al., 2003; Stephan et al., 2004; Sawada et al., 1990).

However, recent large-scale transcriptomic and proteomic analyses change this traditional view. Data from the Human Protein Atlas, which systematically classifies genes encoding extracellular matrix (ECM) and secreted proteins, demonstrate that most ECM-associated genes display tissue-enhanced or group-enriched expression profiles at the mRNA level, whereas only a small fraction exhibit truly ubiquitous expression. These findings indicate that ECM components, including collagens, show pronounced tissue specificity, suggesting that the *in vivo* distribution of type VIII collagen is likely more selective and regulated than previously appreciated (Naba et al., 2016).

Furthermore, evidence from the 29-tissue paired proteome–transcriptome atlas and the mass spectrometry (Naba, 2023) –based “extracellular matrix protein atlas” (McCabe et al., 2023) further supports the tissue-specific expression of collagen family members. These studies reveal marked organ-dependent variation in both expression abundance and subtype composition patterns. Such findings underscore that the tissue-selective expression of extracellular matrix components—particularly collagens—is a critical determinant of local matrix composition, structural organization, and mechanical properties.

Analyses of *Col8a1*/*Col8a2* expression from the Human Protein Atlas (Wang et al., 2019), together with integrative studies of vascular disease–related datasets (Li et al., 2024) indicate that both transcripts and proteins are most abundantly expressed in the cornea, myocardium, large and medium-sized arteries, lung, and kidney. In contrast, expression remains low in parenchymal cell types such as hepatocytes and neurons, yielding an overall pattern of vascular and basement membrane–enriched expression with clear organ-specific biases. Notably, *Col8a2* has been identified as a core structural component of the descemet membrane in the corneal endothelium, and its expression is markedly dysregulated in corneal disorders, including Fuchs’ endothelial corneal dystrophy.

In renal tissue, early studies reported that type VIII collagen is predominantly localized to the glomerular mesangium and segments of small vascular basement membranes. More recent investigation in aged mouse kidneys (Vo et al., 2024) has demonstrated a significant age-dependent increase in *Col8a1* expression within the glomeruli, tubular basement membranes, and renal interstitium. This upregulation is closely associated with structural remodeling of the kidney, including interstitial fibrosis and microvascular rarefaction. Collectively, these findings indicate that type VIII collagen undergoes age-related regional enrichment in the kidney and may contribute to progressive renal remodeling.

From a cellular origin perspective, recent transcriptomic and single-cell RNA-sequencing studies demonstrate that type VIII collagen is not exclusively produced by vascular endothelial cells and vascular smooth muscle cells. Instead, it is also synthesized by organ-resident fibroblasts (Ford et al., 2023)、glomerular mesangial cells, adipose-derived stromal cells (Zhu et al., 2021), tumor-associated fibroblasts (Zhou X. et al., 2024), and other immune-related stromal populations. Notably, under pathological conditions such as inflammation, fibrosis, and tumor microenvironment remodeling, expression of type VIII collagen is further upregulated, highlighting its context-dependent regulation and functional relevance in disease states.

**FIGURE 1:**
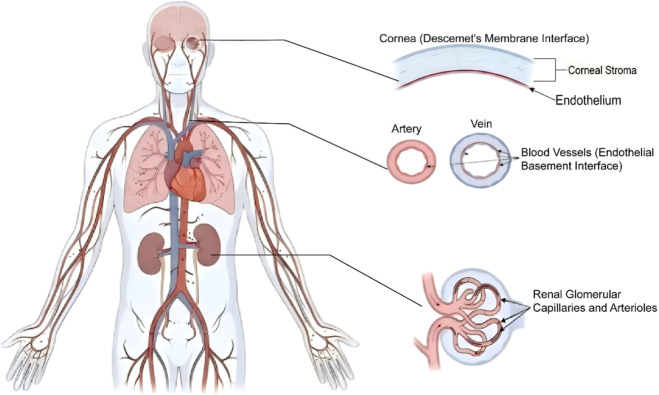
Diagram of the anatomical distribution of type VIII collagen in key tissues of the human body. This figure shows the main physiological positioning of type VIII collagen in endothelial cells and specialized basal membrane tissue. (Abor right) Cornea: In the cornea, type VIII collagen is mainly concentrated in the interface of the posterior elastic layer (Descemet’s Membrane), acting as a key structural barrier connecting the corneal endothelium and the matrix layer (Stroma). (Middle right) Vascular system: In large blood vessels (arteries and veins), type VIII collagen is widely present in the Endothelial Basement Interface under the endothelium, which is mainly secreted by vascular endothelial cells and smooth muscle cells to maintain blood vessels. The structural integrity of the wall. (Lower right) Kidney: In kidney tissue, type VIII collagen is mainly distributed in Renal Glomerular Capillaries and inlet/outlet small arterial walls, and participates in the structural support of the kidney filtration barrier.

By integrating evidence from traditional immunohistochemical approaches with contemporary multi-omics analyses, this study demonstrates that although type VIII collagen is broadly distributed, it exhibits pronounced enrichment and functional specificity in selected tissues and cell types, particularly in the cornea, kidney, and systemic vasculature (Naba, 2023). Accordingly, type VIII collagen should not be regarded as a collagen subtype “lacking tissue specificity.” The *in vivo* distribution profile is summarized in Figure 1.

## 3 Physiological functions of type VIII collagen

Type VIII collagen (COL8) exerts dual and closely integrated functions within the extracellular matrix (ECM), acting both as a bioactive ligand for endothelial and stromal cells and as a structural component of specialized basement membranes. Although COL8 lacks the canonical RGD motif and is therefore not considered a classical integrin ligand, increasing evidence indicates that it interacts with multiple integrin subtypes, including receptors containing β1 (Adiguzel et al., 2013), αvβ3, and αvβ5 (Hou et al., 2000), in a context-dependent manner. Through these interactions, we postulate that type VIII collagen may regulates endothelial adhesion, migration, and vascular remodeling, primarily via activation of FAK/Src and downstream MAPK/ERK and PI3K–AKT signaling pathways. However, the precise binding interfaces, affinity determinants, and structural basis of these interactions remain incompletely defined.

Unlike fibrillar collagens that engage defined integrin receptors, type VIII collagen functions as a provisional matrix protein whose interactions with integrins are dynamically modulated by the tissue microenvironment and pathological remodeling processes (Li et al., 2024). As a provisional matrix ligand, COL8 interacts with collagen-binding integrins α1β1 and α2β1, as well as angiogenesis-associated integrins αvβ3 and αvβ5 (Niland and Eble, 2012) thereby positioning it at the interface of extracellular matrix signaling and vascular biology. Experimental studies in vascular smooth muscle and endothelial cells demonstrate that COL8 regulates angiogenesis and vascular remodeling through β1 integrin–dependent signaling, modulating RhoA activity and MMP-2–mediated cell migration (Adiguzel et al., 2013). Integrins αvβ3 and αvβ5 act as key nodes in angiogenic and Mechan transduction signaling, mediating COL8-dependent activation of FAK, MAPK/ERK, and PI3K–AKT pathways to support endothelial cell survival, directional migration, and barrier integrity. Recent studies in glioma further show that *Col8a1* overexpression promotes tumor cell proliferation and motility while fostering a pro-angiogenic microenvironment, underscoring COL8 as an active signaling ligand *in vivo* (Pang et al., 2023).

**FIGURE 2:**
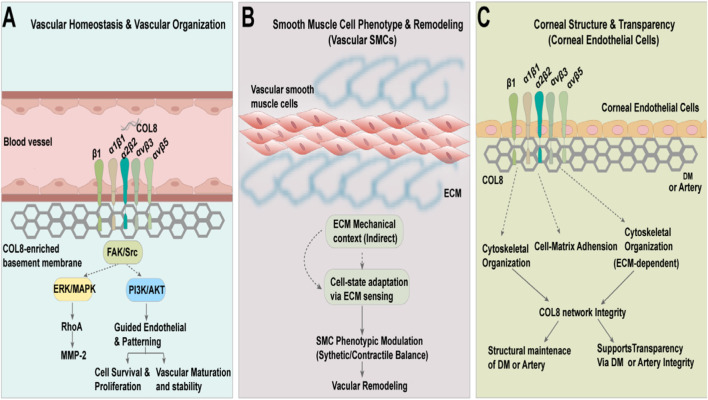
Type VIII collagen–mediated signaling pathways and physiological functions. **(A)** In blood vessels, COL8 is enriched in the endothelial basement membrane and contributes to vascular homeostasis and structural organization. By shaping the COL8-rich ECM, collagen-binding integrins on endothelial cells transduce ECM-dependent cues into intracellular signaling, including FAK/Src with downstream MAPK/ERK and PI3K/Akt pathway modulation. These integrin-associated signals support controlled endothelial cell migration, survival, barrier function, and vessel-level organization and remodeling. **(B)** In the vascular media, COL8 deposition within the subendothelial ECM helps define the local mechanical context. Changes in ECM architecture and stiffness are sensed by collagen receptors and growth-factor signaling systems, indirectly modulating the balance between contractile and synthetic phenotypes of vascular smooth muscle cells, and thereby contributing to structural vascular wall remodeling and adaptation. **(C)** In the cornea, COL8 is a key component of Descemet’s membrane, providing a structural scaffold for corneal endothelial cells. Integrin-mediated cell–matrix adhesion and ECM-dependent cytoskeletal organization help maintain COL8 network integrity and Descemet’s membrane homeostasis, which in turn supports corneal structural integrity and transparency.

Classical ultrastructural and biochemical studies have established COL8 as a structurally specialized basement membrane collagen. In the posterior elastic layer of the cornea, it assembles into a highly ordered hexagonal lattice composed of ∼160-nm pillar-like structures synthesized by corneal endothelial cells. This architecture is maintained throughout life and provides mechanical strength, regulated porosity, and a structural platform for endothelial polarity (Sawada et al., 1990). These lattices interlace with laminin and type IV collagen networks to form a composite basement membrane scaffold. Accordingly, *Col8a1/Col8a2* deficiency in mice results in thinning, fragmentation, and architectural disorganization of the posterior elastic layer, increasing susceptibility to anterior segment abnormalities. In humans, mutations in the *Col8a2* NC1 domain disrupt endothelial polarity and intercellular junctions in early-onset Fuchs’ endothelial corneal dystrophy, underscoring the essential role of COL8 in basement membrane integrity and endothelial homeostasis (Uehara et al., 2021). Beyond the eye, COL8 is enriched within the subendothelial extracellular matrix of arteries and arterioles. Under conditions of disturbed flow, hypertension, and tissue injury, COL8 expression is upregulated, contributing to vascular wall load-bearing capacity, Mechan transduction signaling, and adaptive remodeling. A recent comprehensive review (Li et al., 2024) characterized COL8 as a mechanosensitive, stress-inducible ECM protein that integrates structural reinforcement with integrin- and DDR1-mediated signaling in cardiovascular tissues. In the kidney, age-associated remodeling of glomerular and tubular basement membranes is accompanied by increased COL8 deposition, matrix thickening, and microvascular rarefaction, indicating incorporation of COL8 into an “aged” basement membrane scaffold that alters filtration dynamics and tissue mechanics (Vo et al., 2024). Within the basement membrane collagen family, COL8 is functionally distinct from type IV collagen, which primarily provides a meshwork for structural support, and type XVIII collagen, whose NC1 domain (endostatin) exerts potent anti-angiogenic activity. Consequently, COL8 is increasingly recognized as a hybrid collagen that combines integrin-directed signaling with highly specialized micro-scaffolding functions, thereby uniquely linking extracellular matrix architecture, mechanical signaling, and endothelial biology (Kruegel and Miosge, 2010). A schematic overview of these functions is shown in Figure 2.

## 4 Pathological associations and disease mechanisms of type VIII collagen

### 4.1 In arterial diseases

Type VIII collagen is a critical regulator of arterial injury repair and atherosclerotic progression. Gerth et al. (2007) demonstrated, using *in vitro* cell culture and matrix-coating assays, that COL8 is markedly upregulated during neointimal formation after vascular injury and during atherosclerotic plaque remodeling. In this context, COL8 mediates smooth muscle cell (SMC) adhesion and chemotaxis via integrin-dependent mechanisms and enhances matrix metalloproteinase (MMP) expression, thereby promoting SMC migration and invasion. Complementary findings by Liu et al. (2024), using an *in vitro* fibrous cap model, further elucidated the role of COL8 in plaque stabilization. COL8 expression was significantly increased within the atherosclerotic microenvironment, particularly at stages critical for fibrous cap formation. Mechanistically, COL8 facilitated SMC migration and vascular wall remodeling through specific interactions with oxidized phospholipids.

Together, these studies define that COL8 contributes to the development of vascular disease.

### 4.2 Diabetic nephropathy

Type VIII collagen is markedly upregulated during the progression of diabetic nephropathy. Li et al. (2024) reported significantly increased COL8 mRNA and protein levels in renal tissues from diabetic mouse models and in biopsy specimens from patients with diabetic nephropathy compared with controls. Aberrant accumulation of COL8 in the glomerular basement membrane is closely associated with disease progression, contributing to basement membrane thickening and glomerulosclerosis and thereby impairing renal filtration (Hopfer et al., 2009). Such structural alterations ultimately promote proteinuria, a hallmark clinical feature of diabetic nephropathy Collectively, these findings indicate that COL8 not only serves as a pathological biomarker but also actively participates in disease pathogenesis. Mechanistic insights provided by Loeffler et al. further implicate (Loeffler et al., 2011) COL8 in mesangial cell dysfunction.

Pharmacological inhibition studies revealed that COL8 modulates TGF-β1–induced mesangial cell proliferation in association with the PI3K/Akt signaling pathway. Blockade of PI3K/Akt partially reversed COL8-mediated effects on cell proliferation and related protein expression, suggesting that COL8 influences TGF-β1–driven responses through this pathway. Given the central role of TGF-β1 in promoting extracellular matrix accumulation and renal fibrosis, these data support COL8 as a promising molecular target for therapeutic intervention in diabetic nephropathy.

### 4.3 In tissue fibrosis and organ remodeling

Type VIII collagen is increasingly recognized as a pro-fibrotic extracellular matrix (ECM) component with important roles in cardiovascular, renal, pulmonary, and ocular pathology (Skrbic et al., 2015). In the heart, COL8 contributes not only to matrix accumulation, but also to the maintenance of ventricular wall integrity during remodeling. Integrated single-cell RNA sequencing and spatial transcriptomic analyses have identified a distinct *Col8a1* ^+^ fibroblast subpopulation as a central effector of tissue fibrosis. In injured and failing myocardium, this subset co-expresses WNT2 and WNT4 together with canonical pro-fibrotic mediators, including transforming growth factor-β (TGF-β), platelet-derived growth factors (PDGFs), and fibroblast growth factors (FGFs). Its activation is closely associated with aberrant NF-κB and Hippo–YAP signaling, which synergistically drive excessive collagen deposition and ECM stiffening (Ma et al., 2024).

Analogous *Col8a1*-overexpressing fibroblast or stromal cell clusters have been described in pulmonary and cutaneous fibrosis, where they co-express matrix-associated genes (e.g., COL1A1, COL3A1, LOXL2, and ELN) and localize to regions of increased tissue stiffness and myofibroblast enrichment (Redente et al., 2020). In the kidney, COL8 expression is elevated in experimental diabetic nephropathy and age-related fibrosis (Loeffler et al., 2012). In streptozotocin-induced models, COL8 accumulation in glomerular and tubular basement membranes correlates with epithelial dedifferentiation and epithelial–mesenchymal transition (EMT)–like changes, whereas genetic or pharmacological suppression attenuates mesangial expansion and interstitial fibrosis (Moss et al., 2022). In aged mouse kidneys, increased *Col8a1*/*Col8a2* expression is associated with type I/III collagen deposition, microvascular rarefaction, and declining renal function, implicating COL8 in age-related ECM remodeling (Vo et al., 2024). Metabolic fragments of COL8 have also emerged as informative biomarkers of fibrotic activity. In conditions such as chronic obstructive pulmonary disease and liver fibrosis—where vascular remodeling and basement membrane turnover are prominent—circulating levels of the C-terminal COL8 fragment (C8-C) are markedly elevated, reflecting enhanced collagen degradation and tissue remodeling (Hansen et al., 2016). In ocular fibrosis, increased *Col8a1* expression and deposition in Tenon’s capsule and conjunctiva correlate with postoperative scarring, further supporting a generalized role for COL8 in myofibroblast-rich fibrotic matrices (Seet et al., 2017).

*In vivo* evidence from *Col8a1*/*Col8a2* knockout models demonstrates a dual function for COL8. While wild-type animals exhibit pronounced stress-induced fibrotic remodeling, COL8 deficiency confers protection against matrix accumulation but also leads to maladaptive phenotypes, including premature mortality, ventricular dilation, and cardiac dysfunction (Skrbic et al., 2015). These findings indicate that COL8 not only promotes fibrotic matrix formation but is also essential for preserving tissue integrity under mechanical stress. Consistent with observations in the cornea and kidney, loss of COL8 disrupts basement membrane architecture, underscoring its integral role as a mechanosensitive ECM component that links matrix remodeling to structural stability.

### 4.4 In oncology

High-throughput transcriptomic profiling and pan-cancer analyses indicate that *Col8a1* is among the most consistently upregulated network-forming collagens across a broad spectrum of solid tumors. Elevated *Col8a1* expression is associated with advanced disease stage, extracellular matrix activation, and reduced overall survival in gastric, colorectal, breast, lung, renal, and pancreatic cancers (Zhou et al., 2020). Mechanistically, type VIII collagen facilitates tumor progression through both tumor cell–intrinsic mechanisms and tumor microenvironment–mediated pathways. In gliomas, *Col8a1* is preferentially overexpressed in high-grade tumors and correlates with poor clinical outcomes. Functional studies further show that *Col8a1* promotes glioma cell proliferation, migration, and invasion by activating FAK, AKT, and ERK signaling cascades, whereas *Col8a1* knockdown suppresses tumor growth *in vivo* and reprograms the tumor microenvironment toward a less immunosuppressive state (Liu et al., 2024).

In papillary thyroid carcinoma and breast cancer, overexpression of *Col8a1* promotes epithelial–mesenchymal transition (EMT), upregulates EMT-associated transcription factors and matrix metalloproteinases, and is associated with aggressive clinicopathological characteristics (Liang et al., 2024). In gastrointestinal malignancies, type VIII collagen is strongly linked to cancer-associated fibroblasts (CAFs) and the development of chemoresistance. In colorectal cancer, THBS2^+^ CAFs secrete *Col8a1*, which engages ITGB1 on tumor cells and activates the PI3K–AKT pathway, thereby inducing EMT and conferring resistance to oxaliplatin; pharmacological or genetic inhibition of ITGB1 or AKT reverses *Col8a1*-mediated drug resistance (Zhou X. et al., 2024). Pan-cancer collagen network analyses further identify *Col8a1* as a central node in CAF-enriched, profibrotic tumors, where it co-localizes with other fibrotic collagens (e.g., COL1A1, COL3A1, and COL11A1) and TGF-β–responsive gene signatures (Le et al., 2020). In gastric cancer, multiple independent clinical and experimental studies show that *Col8a1* is significantly upregulated in tumor tissues relative to normal gastric mucosa. High *Col8a1* expression correlates with advanced disease stage, poor prognosis, EMT activation, increased angiogenesis, and chemoresistance. Functional assays demonstrate that *Col8a1* silencing suppresses tumor cell proliferation, invasion, and metastatic potential, while enhancing sensitivity to cytotoxic agents in both *in vitro* models and *in vivo* xenografts, supporting its potential as a therapeutic target and a predictive biomarker for chemotherapy response (She et al., 2022). A similar paradigm is observed in pancreatic ductal adenocarcinoma (PDAC), where tumor- and stroma-derived *Col8a1* establishes autocrine and paracrine loops that promote tumor cell survival and drug resistance; elevated *Col8a1* expression is associated with poor prognosis in resected PDAC (Yan et al., 2022).

Collectively, these studies reveal convergent themes across multiple dimensions of cancer biology. First, type VIII collagen is markedly enriched within pro-fibrogenic, cancer-associated fibroblast (CAF)–dominated tumor microenvironments, where it cooperates with fibrillar collagens and other extracellular matrix (ECM) components to promote matrix stiffening and tumor invasion. Second, *Col8a1* functions as a pro-tumorigenic ligand in cancer cells by activating integrin-dependent FAK–PI3K–AKT signaling and downstream epithelial–mesenchymal transition (EMT) and survival pathways. Third, type VIII collagen–derived fragments and circulating neoepitopes show promise as noninvasive biomarkers that reflect matrix activation, ECM remodeling, and therapeutic response. Together with its established involvement in fibrotic disorders, these findings position type VIII collagen as a critical molecular link between chronic fibrosis, profibrotic tumor microenvironments, and malignant progression.

## 5 Biosynthesis of type VIII collagen

**FIGURE 3:**
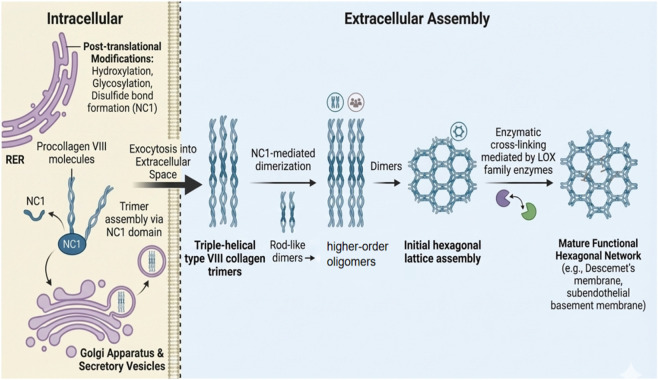
Schematic diagram of intracellular biosynthesis and extracellular hexagonal lattice assembly of type VIII collagen. The figure shows the dynamic process of type VIII collagen (COL8) from synthesis to the formation of a mature extracellular matrix (ECM) network. Intracellular biosynthesis (left): Type VIII pre-collagen polypeptide chains are synthesized in the rough endoplasmic reticulum (RER) and undergo key post-translational modifications, including hydroxylation, glycosylation and disulfide bond formation in the non-collagen domain 1 (NC1). Subsequently, the three polypeptide chains are assembled into a stable trimer (Trimer) through the specific interaction of the C-terminal NC1 domain, and are released to the extracellular space through Exocytosis through the Golgi body and secretory vesicles. Extracellular assembly (right): The secreted three-helical type VIII collagen trimer is first mediated by the NC1 domain to form a reverse parallel rod-like dimer, and then polymerizes into high-order oligomers. These structural units self-assembled to form an initial hexagonal lattice. Finally, under the enzymatic cross-linking effect of the enzyme-mediated by the LOX family, the lattice structure is reinforced to form a mature functional network with mechanical stability. This special hexagonal network structure is the main structural feature of the posterior corneal elastic layer (Descemet’s membrane) and the subcutaneous basal membrane of blood vessels.

Type VIII collagen is a nonfibrillar, short-chain collagen that assembles into specialized extracellular matrix (ECM) architectures, most notably the hexagonal lattice of Descemet’s membrane and subendothelial basement membranes. In contrast to fibrillar collagens, type VIII collagen undergoes a distinctive two-stage assembly process, consisting of intracellular trimer formation followed by extracellular oligomerization into higher-order lattice structures. The biosynthetic pathway of type VIII collagen is described below and illustrated schematically in Figure 3.

### 5.1 Intracellular synthesis, post-translational modification, and trimer assembly

Type VIII collagen is encoded by the *Col8a1* and *Col8a2* genes, which produce the α1(VIII) and α2(VIII) chains, respectively. Newly synthesized α1(VIII) and α2(VIII) polypeptides undergo extensive post-translational modifications before assembling into biologically functional type VIII collagen, with hydroxylation representing a critical regulatory step. Within the lumen of the rough endoplasmic reticulum, specific proline and lysine residues are hydroxylated by prolyl hydroxylase (Yamauchi and Sricholpech, 2012) and lysyl hydroxylase (Sricholpech et al., 2012) respectively, generating hydroxyproline and hydroxylysine. These reactions require Fe^2+^ and vitamin C as essential cofactors, with α-ketoglutarate serving as a co-substrate (Salo and Myllyharju, 2021). Hydroxylation, particularly of proline residues, markedly enhances the thermal stability of the collagen triple helix (Myllylä et al., 1988). Accordingly, vitamin C deficiency impairs hydroxylase activity, disrupts collagen biosynthesis, and results in scurvy, which is clinically characterized by vascular fragility and delayed wound healing (Needleman and Greenwald, 1986). In addition, pathogenic variants in *Col8a2*, such as L450W and Q455K, have been shown to impair hydroxylation, leading to endoplasmic reticulum retention of mutant proteins and failure of proper triple-helix assembly (Gottsch et al., 2005). Collectively, these findings underscore the essential role of hydroxylation in the intracellular processing, secretion, and biological function of type VIII collagen.

In addition to hydroxylation, type VIII collagen undergoes glycosylation, one of the most abundant and structurally diverse post-translational modifications in proteins (Hennet, 2019). This process involves sequential enzymatic reactions within the endoplasmic reticulum and Golgi apparatus mediated by multiple glycosyltransferases and glycosidases (Lowe and Marth, 2003; Schjoldager et al., 2020). In these compartments, galactosyltransferases and glucosyltransferases catalyze the attachment of glucose, galactose, and related disaccharides to 5-hydroxylysine residues (Schegg et al., 2009). Glycosylation of specific hydroxylysine sites within the triple-helical domain is essential for the structural integrity and functional activity of type VIII collagen (Yamauchi and Sricholpech, 2012). Mass spectrometry analyses indicate that corneal-derived *Col8a2* is glycosylated at Hyl-450 and Hyl-455, whereas vascular-derived *Col8a1* exhibits lower overall glycosylation, suggesting tissue-specific regulation (Basak et al., 2016). Although the precise functional consequences remain incompletely defined, available evidence indicates that glycosylation may influence collagen supramolecular organization, fiber orientation, and cell–matrix interactions (Torre-Blanco et al., 1992). Variation in glycan composition and site occupancy across tissues likely modulates protein stability, chain selection, and biological activity. Trimer assembly initiates at the C-terminal NC1 domain, which functions as the folding nucleus and determines trimer stoichiometry (Gansner and Gitlin, 2008). High-resolution structural studies further demonstrate that the NC1 domain adopts a C1q-like β-sandwich fold that mediates chain-specific recognition and stabilizes trimer formation (Kvansakul et al., 2003). Despite these insights, the complete *in vivo* trafficking route of type VIII collagen—from endoplasmic reticulum and Golgi processing through vesicular transport, secretion, and extracellular lattice assembly—has not been fully delineated and is largely inferred from other collagen types. Notably, genetic ablation of *Col8a1* or *Col8a2* in mice results in thinning and structural abnormalities of Descemet’s membrane, providing *in vivo* confirmation that proper intracellular trimer assembly is essential for basement membrane integrity (Stunf, 2022).

### 5.2 Extracellular oligomerization and hexagonal lattice formation

Unlike fibrillar collagens, type VIII collagen retains its N- and C-terminal non-collagenous domains after secretion, which are essential for supramolecular assembly (Karamanos et al., 2021). Ultrastructural analyses of human and bovine Descemet’s membranes by electron microscopy reveal highly ordered hexagonal lattices composed of type VIII collagen, characterized by repeating pillar-like structures approximately 160 nm in length that form a honeycomb-like, two-dimensional network (Sawada et al., 1990). Biochemical and recombinant protein studies further demonstrate that assembly follows a hierarchical process: secreted trimers first associate into basic oligomers through NC1 domain–mediated interactions, which then organize into higher-order tetrahedral complexes (Illidge et al., 2001).

α1(VIII) homotrimers assemble into tetrahedral units consisting of four trimers arranged symmetrically around a central NC1 core, a structure identified as the minimal supramolecular building block of the type VIII collagen network (Stephan et al., 2004). Through lateral associations and angle-specific interactions, these tetrahedra further organize into planar hexagonal lattice sheets, which subsequently stack and undergo cross-linking to form the dense and mechanically resilient three-dimensional architecture characteristic of Descemet’s membrane (Sawada et al., 1990). Thus, despite its relatively short collagenous domains, type VIII collagen constructs robust extracellular matrix structures through specialized oligomerization rather than classical fibril formation.

### 5.3 Integration into specialized basement membrane networks

Type VIII collagen lattices co-localize with and integrate into basement membranes together with canonical components, including type IV collagen, laminins, nidogen, and heparan sulfate proteoglycans (Song et al., 2021). This integration confers biomechanical stability, regulates hydration and molecular diffusion, supports endothelial cell polarity and adhesion, and enhances resistance to deformation and mechanical stress (Ali et al., 2016; Saikia et al., 2018). Histological and ultrastructural analyses subdivide the postelastic layer of Descemet’s membrane into two regions: an anterior banded zone (ABZ) enriched in hexagonal type VIII collagen lattices, and a posterior non-banded zone (PNBZ) containing a mixed network of type VIII and type IV collagens, laminins, and proteoglycans (de Oliveira et al., 2020). Ongoing synthesis of type VIII collagen throughout adulthood contributes to age-associated thickening of the PNBZ (Jun et al., 2006).

### 5.4 Pathological consequences of impaired ECM assembly

Pathogenic variants in *Col8a1* or *Col8a2*, particularly within the NC1 domain, disrupt trimerization and hexagonal lattice assembly, leading to compromised endothelial polarity, impaired barrier function, and altered ZO-1 distribution (Gottsch et al., 2005). These defects are central to early-onset Fuchs’ endothelial corneal dystrophy (FECD). In addition, oxidative stress, inflammation, and age-related declines in endothelial secretory capacity further impair type VIII collagen assembly, underscoring its critical role in maintaining basement membrane structure and function (Ong Tone et al., 2021).

## 6 Research and applications of type VIII collagen

Owing to its distinctive structural properties and biological functions, type VIII collagen shows considerable promise in biomedical applications, particularly in ophthalmic disease therapy, tissue repair and regeneration, and regenerative medicine. In parallel with growing basic research, a number of patents related to type VIII collagen have been filed internationally, reflecting increasing translational interest.

For example, the patent entitled “Method for Biosynthetic Human Structural Material Type VIII Collagen”filed by Shanxi Jinbo Biopharmaceutical Co., Ltd (Zhang et al., 2024). reports the successful development of recombinant type VIII collagen through optimized sequence design and expression strategies, addressing key limitations of native collagen, including immunogenicity and challenges in large-scale production. This technological advancement provides a foundation for the manufacture of high-grade medical devices and biomedical products. The patent further outlines potential applications of type VIII collagen in bioengineered dressings, biomimetic materials, and cosmetic and reconstructive medicine, highlighting its feasibility and versatility for clinical translation.

### 6.1 Regenerative medicine and tissue engineering

Type VIII collagen has attracted increasing interest in regenerative medicine and tissue engineering because of its distinctive capacity to promote endothelial cell adhesion, migration, and microenvironmental organization—functions that are not fully recapitulated by fibrillar collagens or synthetic scaffolds. Experimental studies show that modification of collagen matrices with hyaluronic acid oligosaccharides accelerates endothelialization and directs cell alignment (Jia et al., 2022), Inspired by this research, it is conceivable that future scaffold designs incorporating hyaluronic acid oligosaccharide modifications could be engineered to enrich or mimic type VIII collagen, thereby providing critical biochemical and topographical cues that facilitate early vascular integration and microvascular pattern formation. In corneal applications, bioengineered matrices and three-dimensional printed constructs incorporating type VIII collagen exhibit excellent biocompatibility and structural fidelity (Orash et al., 2022; Guérin et al., 2021), enabling corneal epithelial and endothelial cells to restore adhesion, polarity, and matrix organization with minimal inflammatory response. Notably, these engineered substitutes more closely reproduce the lattice-like ultrastructure of Descemet’s membrane than conventional collagen hydrogels, highlighting their potential for treating corneal endothelial disorders and postoperative stromal defects. Despite these advances, several challenges hinder clinical translation of COL8-based scaffolds, including the difficulty of recapitulating native post-translational modifications required for proper folding, instability of supramolecular assembly in recombinant systems, and the need for scalable, Good Manufacturing Practice (GMP)–compliant production workflows.

### 6.2 Diagnostic applications

Accumulating evidence suggests that type VIII collagen and its proteolytic fragments may serve as clinically informative biomarkers for disorders marked by extracellular matrix remodeling, dysregulated angiogenesis, or basement membrane injury. Competitive ELISA assays targeting type VIII collagen have revealed significantly elevated serum levels in patients with chronic obstructive pulmonary disease (COPD) and several malignancies, including lung squamous cell carcinoma, breast cancer, and colorectal cancer, whereas no significant change has been observed in idiopathic pulmonary fibrosis. These data support the potential utility of type VIII collagen as a diagnostic and disease-monitoring biomarker in selected cancers, although its value as a surrogate for response to anti-angiogenic therapy remains to be established (Hansen et al., 2016). More recently, COL8-related blood biomarkers have progressed from proof-of-concept studies to cohort-based evaluations of prognostic significance. In cholangiocarcinoma, baseline serum levels of the *Col8a1* NC1 domain are significantly higher than those in healthy controls and independently predict overall and progression-free survival. Moreover, an early decline after the first chemotherapy cycle is more frequent among patients achieving stable disease or partial response, indicating potential utility for treatment-response monitoring (Kaps et al., 2025). By contrast, studies in metastatic pancreatic and colorectal cancers indicate that COL8-derived fragments (e.g., C8-C or vastatin) reflect tumor stromal activity but provide limited incremental prognostic value beyond established collagen turnover markers and conventional tumor biomarkers (Wang et al., 2021). In diabetic nephropathy, elevated circulating levels of type VIII collagen degradation products (e.g., C8-C) correlate with disease progression, supporting their use as early indicators of renal injury (Gerth et al., 2007). Despite these promising findings, clinical implementation of COL8-based biomarkers is constrained by insufficient analytical specificity relative to other collagens—particularly types IV and X—highlighting the need for improved assay design and validation.

### 6.3 Therapeutic development

Therapeutic strategies targeting COL8-related disorders are rapidly advancing and encompass gene editing, cell-based therapies, tissue engineering, biomaterials development, and molecular pharmacology. Gene editing represents the most direct approach for correcting pathogenic *Col8a2* variants associated with early-onset Fuchs’ endothelial corneal dystrophy (FECD). CRISPR/Cas9-mediated disruption of mutant start codons has been shown to restore endothelial cell polarity and reduce corneal edema in murine models, demonstrating the feasibility of allele-specific intervention (Uehara et al., 2021). Complementing gene-based approaches, cell therapy has reached early clinical application through transplantation of cultured corneal endothelial cells in combination with Rho kinase inhibitors, with initial trials reporting sustained corneal clarity and low immunogenicity (Okumura et al., 2018). In addition, mesenchymal stem cell–derived exosomes have been shown to deliver anti-inflammatory microRNAs that modulate NF-κB signaling and partially restore COL8 homeostasis, yielding therapeutic benefit in experimental models of COL8-deficient FECD (Liu et al., 2008; Raffetto and Khalil, 2008). At the biomaterials level, recent reviews in corneal bioengineering underscore growing interest in engineered substrates and tissue-engineered alternatives for Descemet membrane endothelial keratoplasty (DMEK), driven primarily by donor tissue shortages (Zwingelberg et al., 2025). Although collagen-based scaffolds for corneal stromal and endothelial repair have been extensively explored (Orash et al., 2022; Namli et al., 2025; Chi et al., 2023; Gingras et al., 2023), no peer-reviewed studies to date have reported type VIII collagen–based scaffolds or three-dimensional printed constructs that recapitulate the native hexagonal lattice of Descemet’s membrane and support durable endothelial function *in vitro* or *in vivo*. Despite substantial progress in bioprinting and scaffold engineering, major translational barriers remain, including access to native or recombinant COL8 with appropriate post-translational modifications, reproducible supramolecular assembly, and scalable Good Manufacturing Practice (GMP)–compliant production. Accordingly, COL8-based biomaterials should be regarded as a promising yet unvalidated avenue for future translational research.

Despite growing structural insights into the NC1 domain of type VIII collagen, no pharmacological agents specifically targeting this domain are currently available. To date, the only direct strategy aimed at COL8 involves CRISPR/Cas9-mediated allele disruption in a mouse model of early-onset Fuchs’ endothelial corneal dystrophy (FECD), which effectively arrested endothelial degeneration. Other therapeutic approaches, such as Rho kinase (ROCK) inhibitor–assisted transplantation of ex vivo–expanded corneal endothelial cells, have indirectly restored a COL8-containing basement membrane milieu without directly modulating COL8. Consequently, COL8-directed therapy remains at a nascent stage, with most efforts focused on structural characterization, disease genetics, and proof-of-concept gene editing.

Although interest in type VIII collagen has expanded across diverse pathological contexts—including corneal endothelial disorders, vascular remodeling, and fibrosis—its clinical translation is hindered by several practical challenges. Chief among these is the pronounced context dependence of COL8 function; for example, in atherosclerosis it contributes to plaque stabilization yet may also exacerbate luminal narrowing, necessitating carefully timed and spatially restricted interventions to avoid adverse effects. Moreover, the absence of highly specific COL8 inhibitors constrains pharmacological development, and the structural homology within the collagen superfamily raises concerns about off-target effects. In parallel, the technical difficulty of producing biologically active full-length type VIII collagen limits its scalability for regenerative applications.

Accordingly, future translational advances are unlikely to arise from single-target blockades alone. Instead, emphasis should be placed on precision strategies—such as targeted delivery platforms (e.g., siRNA nanocarriers) and advanced gene-editing technologies—that enable spatiotemporal control of COL8 expression. Key applications, challenges, and prospective directions are summarized in Table 1.

**TABLE 1:** Summary table of the main applications, challenges, and future directions of type VIII collagen.

Clinical area	Evidence level	Current challenges	Future directions
Ophthalmic diseases (FECD, corneal transplantation)	*Col8a2* established as a causative gene for FECD; A COL8A2 Q455K point-mutation mouse model exhibits endothelial cell loss and descemet’s membrane abnormalities that closely resemble those observed in human FECD, thereby supporting the pathogenic role of this mutation (Jun et al., 2012)	Mutant protein retention in the endoplasmic reticulum activates the unfolded protein response (UPR); no effective non-surgical therapies currently available	Allele-specific silencing using siRNA or CRISPR systems; development of nanostructure-based artificial corneal endothelium
Cardiovascular disease (atherosclerosis)	Genetic ablation reduces vascular remodeling; marked upregulation observed in human atherosclerotic plaques (Li et al., 2024)	Dual role in plaque stabilization and luminal narrowing complicates therapeutic timing and strategy	Exploration of COL8 as a novel therapeutic target for atherosclerosis prevention and intervention
Oncology (breast and colorectal cancer)	High expression correlates with poor prognosis; *in vitro* studies confirm roles in invasion and migration (Liu et al., 2024)	Absence of highly selective inhibitors targeting COL8–integrin interactions	Development as a diagnostic and prognostic biomarker for ECM-driven malignancies
Fibrotic diseases (renal, hepatic, pulmonary)	Consistently upregulated in animal models; causal mechanisms remain under active investigation (Skrbic et al., 2015)	Functional redundancy with COL1 and COL3; compensatory effects limit single-target therapies	Combination strategies integrating COL8 inhibition, targeted delivery, and TGF-β pathway modulation
Tissue engineering (biomaterials)	Proof-of-concept studies demonstrate biocompatibility of recombinant COL8 coatings (Guérin et al., 2021)	Difficulty reproducing native post-translational modifications; unstable supramolecular assembly; lack of scalable MP pipelines	Development of bioinspired hydrogels incorporating COL8-derived functional peptides and 3D-printed scaffolds

## 7 Summary and discussion

Type VIII collagen is an essential component of the extracellular matrix and plays a pivotal role in maintaining tissue homeostasis and regulating pathological processes, owing to its distinctive short-chain structure and broad tissue distribution. Physiologically, it contributes to fundamental functions across multiple systems, from preserving corneal endothelial integrity to dynamically modulating vascular remodeling. Pathologically, dysregulated expression of type VIII collagen has emerged as a key determinant in the progression of diverse disorders, including vascular disease, renal fibrosis, and tumor microenvironment remodeling.

Current evidence supports both the diagnostic and translational relevance of type VIII collagen. Circulating markers such as C8-C show potential value in disease detection and monitoring (e.g., in diabetic nephropathy), while gene-based interventions, including CRISPR-mediated correction of pathogenic variants, highlight opportunities for therapeutic development in corneal disorders. Nevertheless, major knowledge gaps remain. Critical unanswered questions include the context-dependent roles of type VIII collagen in tumor angiogenesis, tissue-specific heterogeneity in its interaction networks, and the development of biocompatible biomaterials capable of faithfully recapitulating its native structure and function across species.

Future progress is likely to be driven by the integration of emerging technologies such as single-cell omics, organoid modeling, advanced biomaterials, and precision gene-editing platforms. These advances are expected to accelerate (i) the development of highly specific molecular probes for early and accurate diagnosis, (ii) the engineering of dynamic biomimetic scaffolds that reproduce the spatiotemporal organization of native extracellular matrices, and (iii) the design of structure-guided small-molecule modulators to selectively disrupt disease-associated signaling pathways. Collectively, these efforts will deepen mechanistic insight into matrix–disease relationships and promote the translation of “matrix medicine” from basic discovery to clinical application, with particular relevance to vascular degenerative disorders and inherited corneal diseases.

## Data Availability

The original contributions presented in the study are included in the article, further inquiries can be directed to the corresponding authors.
